# A systematic review of risk and protective factors of mental health in unaccompanied minor refugees

**DOI:** 10.1007/s00787-020-01678-2

**Published:** 2020-11-09

**Authors:** Edgar Höhne, Anna Swantje van der Meer, Inge Kamp-Becker, Hanna Christiansen

**Affiliations:** 1 Department of Child and Adolescent Psychiatry, Psychosomatics and Psychotherapy, Faculty of Human Medicine, Philipps-University Marburg, Schützenstraße 49, 35039 Marburg, Germany; 2 Department of Clinical Child and Adolescent Psychology and Psychotherapy, Philipps-University Marburg, Gutenbergstraße 18, 35037 Marburg, Germany

**Keywords:** Unaccompanied minor refugees, Mental health, Risk factor, Protective factor

## Abstract

In recent years, there has been a rising interest in the mental health of unaccompanied minor refugees (UMR), who are a high-risk group for mental disorders. Especially the investigation of predictive factors of the mental health of young refugees has received increasing attention. However, there has been no review on this current issue for the specific group of UMR so far. We aimed to summarize and evaluate the existing findings of specific risk and protective factors to identify the most verified influences on the mental health of UMR. Therefore, we conducted a systematic literature search. Study designs were limited to quantitative cross-sectional and longitudinal designs. Eight databases were searched in four different languages and article reference lists of relevant papers were screened. 27 studies were included (*N* = 4753). Qualitative synthesis revealed the number of stressful life events to be the most evaluated and verified risk factor for mental health of UMR. A stable environment and social support, on the other hand, can protect UMR from developing poor mental health. Besides that, several other influencing factors could be pointed out, such as type of accommodation, family contact, gender and cultural competences. Because of the large heterogeneity of outcome measures, quantitative synthesis was not possible. This review helps to improve our understanding of determinants of UMRs mental health and thus to provide more targeted treatment. Furthermore, it provides information on how to prevent the development of mental health problems by specifying factors that can be modified by different health and immigration sectors in advance. Further research is needed focusing on the interaction between the various predictive factors.

**Electronic supplementary material:**

The online version of this article (10.1007/s00787-020-01678-2) contains supplementary material, which is available to authorized users.

## Introduction

According to the United Nations High Commissioner for Refugees (UNHCR), by the end of 2019, the number of people forcibly displaced due to war, conflicts, persecution or human rights violation had grown to almost 80 million [1]. This is the highest level of displacement on record. More than half (58%) of the refugees came from Syria, Venezuela, Afghanistan or South Sudan. About 40% of all refugees are minors, with 153,300 of them being reported as unaccompanied or separated from their families at the end of 2019 [1]. According to the European Union (EU) asylum acquis, an unaccompanied minor refugee (UMR) is a non-EU national or stateless person under the age of 18 who arrives on the territory of the EU States unaccompanied by an adult responsible for him/her, and for as long as s/he is not effectively taken into the care of such a person. Around 90% of the UMR who arrived in Europe in 2018 were male and between 15 and 17 years old, whereas the proportions among accompanied minor refugees (AMR) are more balanced in terms of gender and age [2]. The majority of UMR had to leave their country because of war and/or political or religious persecution followed by a long and stressful flight through multiple countries while being separated from their family members. They had to leave their home, their friends and family and leave everything behind for an uncertain future while still being a minor, handling the usual emotional developmental issues of that age. This challenge of handling age-specific and migration-specific struggles can be described as a double disruption in child development [3]. Adolescent developmental tasks as for example to negotiate relationships with parents, the demand of increasingly mature roles and responsibilities are triggered and pushed by the experiences made on flight. But there are many other challenges: to define a personal sense of identity, to develop stable and productive peer relationships, to adjust to new bodily and sexual feelings and to adopt a personal value system. But, all these tasks may be hampered by the challenges they face before and during the flight and by the fact that UMR stay in a foreign culture including many aspects that differ significantly from his or her home culture.

Almost every UMR endures at least one stressful life event (SLE) before or during the escape from their country of origin [4–6]. The SLEs most frequently mentioned were having experienced life-threatening events, physical violence and the loss of close family members. On arrival in the host country, UMR have to cope with multiple strains from the past while learning a new language and adjusting to a new culture, education system and social environment [7]. On top of that, they have to fear a long and difficult asylum-process as well as social discrimination. The fact that they have to deal with all these burdens without any protection or shelter from their families makes it even harder. Therefore, it is quite clear that UMR belong to one of the most vulnerable groups concerning mental health problems. Given that, it is remarkable that about half of the UMR show great resilience and do not develop clinically relevant mental disorders [4, 8, 9]. So far, the present literature lacks explanations for this astonishing resilience. Altogether, UMR are a very special and vulnerable group of refugees and results from studies concerning adult refugees or AMR cannot necessarily be transferred [10].

### Mental health of UMR

Due to the rising numbers of UMR in recent years, research of mental health in this field has been increasing. Kennedy and colleagues reported that after their arrival, on average, immigrants were physically healthier than native born people [11]. They called this finding Healthy-Immigrant-Effect. However, these findings are in contrast to recent findings of the physical and mental health of UMR [4, 12, 13]. For example, one study found higher scores for infectious diseases and other medical conditions such as suffering from headaches and back pain in a refugee population after their arrival [12]. In an epidemiological study of mental disorders in UMR, 41.9% of the UMR met the DSM-IV criteria for a mental disorder, most of them suffering from posttraumatic stress disorder PTSD (30.6%), major depression (9.4%), agoraphobia (4.4%) and general anxiety disorder (3.8%) [4]. In a review on psychological distress of refugee children these findings were confirmed with prevalence rates for PTSD in UMR ranging from 19 to 54% and for depression from 3 to 30%, whereas prevalence of PTSD in non-displaced children only ranged from 2 to 9% [13]. A meta-analysis on depression of children and adolescents reported a prevalence in non-displaced children of 5.9% [14]. Thus, there is strong evidence that UMR have a higher risk of developing mental health problems than non-displaced children. However, studies investigating mental health parameters in UMR compared to AMR are rare. One comparative study [15] could not find differences in the mental health status of these two groups, whereas another study reported significantly more symptoms of depression, PTSD and other anxiety disorders in UMR than AMR [16]. Furthermore, being unaccompanied correlated with a higher risk of exposure to violence and other SLE [16–18]. Considering the high risk of developing mental health problems for UMR, it is necessary to investigate the factors influencing their mental wellbeing.

### Risk and protective factors

According to a study examining mental health and wellbeing as well as behavioral parameters in a large sample of German children and adolescents, a rising number of risk factors increases the prevalence of mental health problems in minors [19]. However, findings suggest that this impact can be attenuated by a rising number of protective factors [19]. In two combined reviews from 2012 concerning the mental health of refugee children, multiple predictive factors were summarized such as age, number of traumatic life events or gender and family support [18, 20]. Unfortunately, these two reviews did not differentiate between UMR and AMR within their analysis of predictive factors despite the fact that these groups differ essentially regarding family support, migration process and living conditions [10]. Due to the rising interest in the mental health of UMR in general, the investigation of UMR-specific predictive factors has received increasing attention in recent years. Corresponding results of these studies are of wide range and partially inconsistent, which is why there is an urgent need for a systematic evaluation. This review will be the first to provide an overview of all published quantitative studies investigating predictive factors of UMRs mental health. Further, the level of verification of the reported predictive factors will be inspected. Based on these findings, this review will identify the most verified influences on the mental health of UMR to help establish effective and early interventions of this most vulnerable refugee population.

## Methods

### Design

Reporting follows the preferred reporting items for systematic reviews and meta-analyses (PRISMA) guidelines [21]. There was no protocol and registration proceeded in advance.

### Criteria for inclusion

For this review, we included all original studies with unaccompanied minor refugees up to the age of 21. Those with wider age categories were only eligible for inclusion if the mean age was 19 years or younger. Studies with mixed samples, including some accompanied or non-displaced children, were only included if the results were stratified to clarify which findings related to the unaccompanied minors. Furthermore, quantitative results of potential risk and protective factors for any mental health outcome had to be reported. Only original and published papers were eligible for inclusion. Meta-analyses and reviews were not included in our review. Papers had to be written in English, German, Spanish, French or Dutch and the sample size of each included study had to be at least *N* = 20. Study designs were limited to cross-sectional and longitudinal designs.

### Search strategies

A widespread literature search was carried out for studies that were reported until March 2019. Publication dates were unrestricted. The following databases were searched systematically: PsycINFO, PSYINDEX, Web of Science, PubMed, ERIC, Cochrane Library and PubPsych. Additionally, Google Scholar and article reference lists of relevant studies were searched as well. The terms were used separately for each language. Terms within a category were linked with “or” whereas terms between categories were linked with “and”, such as (resilien* OR protective* OR resource* OR risk* OR stressor* OR protector*) AND (minor* OR youth* OR adolescent* OR teenage* OR child*) AND unaccompanied* AND (refugee* OR immigrant* OR asylum-seek* OR displaced OR migrant*). Adaptions to the terms were implemented according to the search style of each database. Details on the search terms used for each language are illustrated in the Supplemental Appendix A.

### Study selection

Two reviewers independently screened abstracts and full-texts against the pre-specified criteria for inclusion. Disagreement at any stage of the study selection process were resolved by discussion or by involving a third reviewer. Subsequently, the two reviewers performed a standardized data extraction to gather relevant information systematically from each eligible study. For each study, data relating to study details, methodological information, population characteristics, outcome measures and predictive factors were extracted. A third reviewer checked all extracted data for completeness and accuracy. If multiple studies were produced from the same primary data, new information had to be provided by the additional study to be included. Otherwise the most relevant study to answer the review aims was included.

### Synthesis of results

As mentioned, the potential risk and protective factors are numerous. In the present literature, there are mainly two ways to classify risk and protective factors of the mental health in UMR. Some authors discriminate the factors by the time of their appearance in pre-, peri- or post-risk factors. The categorization applies to protective factors as well. Another way to classify the various predictive factors is by the ecological system theory [22]. This framework constitutes the effect of different factors in child development by the allocation of different sources of influence (e.g., individual, family, community and societal). Since most predictive factors cannot be explicitly assigned to the theory- or time-based categories above, we decided to categorize the investigated factors solely according to content-based criteria. For example, the factors living in foster care and living with a family member were assigned to the category accommodation. As a result, we defined eleven different categories which are largely congruent with the subcategories described in two well-established systematic reviews in this field [18, 20]. Nevertheless there still remains a little overlap between the newly defined categories in terms of factor allocation.

### Risk of bias

We evaluated the quality and the risk of bias of the included studies using the AXIS tool, a standardized critical appraisal tool to assess the quality and risk of bias in cross-sectional studies [23]. The risk of bias was assessed by two independent reviewers. Again, the reviewers solved disagreement by consensus. We condensed the information into a final risk of bias rating for each individual study using three distinct categories: low (18–20), medium (14–17) or high (0–13) risk of bias. In addition to the AXIS tool, we carefully screened each included study for selective outcome reporting. To reduce the risk of selective reporting, this review will report the statistically non-significant predictors of the examined studies as well. Further, if potential predictors were assessed and described in the reporting of the measures of the included studies but are not subsequently reported in the results, they will be labeled as non-significant as well. The level of verification for each predictor will be indicated in accordance with their number of (non-)replications and in terms of sample sizes and study designs.

## Results

**Fig. 1:**
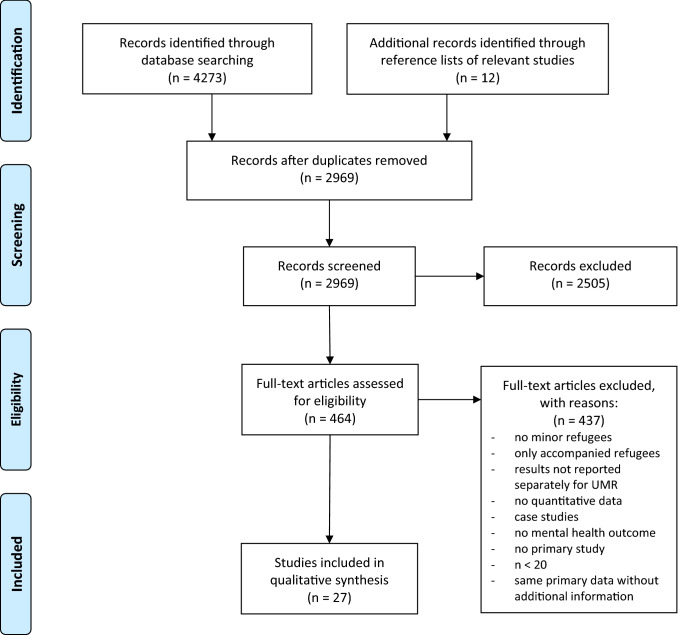
Flow diagram according to PRISMA-Guidelines

The literature search led to 4273 potentially relevant studies, with 1304 duplicates. The remaining 2969 studies were screened by their title and abstract, leading to the exclusion of 2505 studies as they did not meet inclusion criteria. The remaining 464 papers underwent full text screening for eligibility. Finally, 27 studies were included in this review, one of them being a graduate thesis that met all criteria and was open source. The included studies covered 9735 participants in total. Next, we removed overlapping sample sizes of investigators, who had reassessed the same sample in different studies (with different outcomes). Then we excluded the number of non-UMR-participants of the studies which compared UMR to non-UMR. The final number of UMR investigated was 4753, with a wide range of *N* = 18 to *N* = 1110 participants with a mean sample size of *n* = 176. For a detailed display of the results of the literature research, see the flow diagram according to the PRISMA-Guidelines [21] in Fig. 1.

Follow-ups varied between 4 and 32 months. Some studies were conducted right after immigration, others several years after resettlement. The included studies were published between 1987 and 2019. The results were mainly reported as correlations between potential risk or protective factors and mental disorders measured by questionnaires. Some authors additionally used (semi-)structured interviews to quantify the results. The main issues of the Risk of Bias highlighted by the AXIS tool were (a) the missing justification of the sample size, (b) a lack to address or categorize non-responders and (c) the failure to declare any sources of funding or the authors’ conflicts of interests. Especially the comparably older studies struggled to comply with the AXIS conditions (see Supplementary Appendix B).

Mental health outcome measures were widespread and ranged between depression, PTSD, anxiety, internalizing and externalizing behavioral problems. To simplify the display of the results, we summarized the different mental health outcomes to one global psychological distress outcome. A similar approach was used before in two systematic reviews about risk and protective factor of mental health in minor refugees [18, 20]. For a detailed description of the outcome measures and other study characteristics see Table 1. In the following, the results of the literature search will be reported according to contend-related categories. Further, potential inconsistencies or deficiencies within and between the studies will be discussed. Table 2 summarizes the investigated factors.

**Table 1:** Study characteristics of included studies

Author, year	CNr	Country	Design	Study population	*n*	Age	Outcome measure	Qual	RoB
Bean et al., 2007a	[5]	The Netherlands	Cross	Representative	1110	12–18; Ø 15.8	HSCL-37 A, RATS	18/20	Low
Oppedal and Isdoe, 2015	[6]	Norway	Cross	Representative	895	Ø 18,6	CES-D, IWRTE	18/20	Low
Keles et al., 2015	[7]	Norway	Cross	Representative	895	Ø 18.6	CES-D	19/20	Low
Keles et al., 2018	[8]	Norway	Long	Representative	918	Ø 19.0	CES-D	19/20	Low
Rücker et al.,2017	[9]	Germany	Cross	Almost all male	52	12–21; Ø 16,2	RHS-15	14/20	Medium
Bean et al., 2007b	[16]	The Netherlands	Long	Representative	582	11–18; Ø 16.5	HSCL-37 A, RATS, CBCL, TRF	18/20	Low
Bronstein et al., 2013	[24]	UK	Cross	Afghan, all male	222	13–18; Ø 16.3	HSCL-37 A	19/20	Low
Hodes et al., 2008	[25]	UK	Cross	Representative	78	13–18; Ø 17	HTQ, IES, BDSR	17/20	Medium
Hollins et al., 2007	[26]	UK	Cross	Kosovan, Albanian; almost all male	99	13–18; Ø 16	GHQ-28, UCLA LS	16/20	Medium
Sourander, 1998	[27]	Finland	Cross	80% Somalian	46	11–20, Ø 17.4	CBCL	13/20	High
Smid et al., 2011	[28]	The Netherlands	Long	Representative	920	12–18	HSCL-25, RATS	19/20	Low
Bronstein et al., 2012	[29]	UK	Cross	Afghan, all male	222	13–18; Ø 16.3	RATS	19/20	Low
Derluyn et al., 2009	[30]	Belgium	Cross	Representative	124	11–18, Ø 16.6	HSCL-37 A, RATS	19/20	Low
Jakobsen et al., 2017	[31]	Norway	Long	All male	138	15–18; Ø 16,2	HSCL-37A, CPSS	19/20	Low
Jensen et al., 2015	[32]	Norway	Cross	Representative	93	10–16; Ø 13.8	HSCL-37A, CPSS	16/20	Medium
Jensen et al., 2014	[33]	Norway	Long	Representative	75	13–20; Ø 16.5	HSCL-37A, CPSS	19/20	Low
Müller et al., 2019	[34]	Germany	Cross	Almost all male	68	Ø 16.3	CATS, HSCL-37A	17/20	Medium
Porte and Torney-Purta, 1987	[35]	USA	Cross	Indochinese	82	12–19; Ø 16,4	CES-D	13/20	High
Völkl-Kernstock et al., 2014	[36]	Austria	Cross	African UMR	40	15–18, Ø 16.9	UCLA PTSD, SCWP	16/20	Medium
Reijneveld et al.,2005	[37]	The Netherlands	Cross	~ 90% African	122	14–18, Ø 16.2	HSCL-25, RATS	19/20	Low
Vervliet et al., 2014	[38]	Belgium	Long	Representative	103	14–18, Ø 16	HSCL-37A, RATS	18/20	Low
Entholt et al., 2018	[39]	UK	Cross	Representative	35	16–21	SCID-IV, RATS	15/20	Medium
Stotz et al., 2015	[40]	Germany	Cross	All male	32	11–20; Ø 18.3	UCLA PTSD, TRGI, SVQ	14/20	Medium
Müller-Bamouth et al., 2016	[41]	Germany	Cross	All male	49	13–21; Ø 17.4	AAS-C, UCLA PTSD	16/20	medium
Geltman et al., 2005	[42]	USA	Cross	Sudanese UMR	304	Ø 17.6	HTQ, CHQ	19/20	Low
Huemer et al., 2013	[43]	Austria	Cross	African UMR	41	15–18; Ø 16.9	WAI, YSR	16/20	Medium
Sierau et al., 2019	[44]	Germany	Cross	All male	105	14–19; Ø 17,3	LEC-5, PCL-5, PHQ-9, GAD-7, SSS-8, SDQ	19/20	Low

*CNr* citation number, *Design* study design, *cross* cross-sectional design, *long* longitudinal design, *Qual.* quality appraisal using the AXIS tool, *RoB* risk of bias, *HSCL-37* *A* Hopkins Symptoms Checklist-37 for Adolescents, *RATS* reactions of adolescents to traumatic stress Questionnaire, *CBCL* Child Behavior Checklist, *TRF *Teacher Report Form, *CES-D* Center for Epidemiologic Studies-Depression Scale, *HTQ* Harvard Trauma Questionnaire, *IES* Impact of Event Scale, *BDSR* Birleson Depression Self-Rating Scale, *GHQ-28* General Health Questionnaire, *UCLA* University of California at Los Angeles Loneliness Scale, *RHS-15* Refugee Health Screener, *HSCL-25* Hopkins Symptoms Checklist-25, *UCLA PTSD* University of California at Los Angeles Posttraumatic Stress Disorder Reaction Index, *SCWP* Scales for Children Afflicted by War and Persecution, *CPSS* Child PTSD Symptom Scale, *CATS* Child an Adolescent Trauma Screen, *SCID-IV* Structured Clinical Interview for DSM-IV, *SDQ* Strengths and Difficulties Questionnaire, *AAS-C* Appetitive Aggression Scale for children, *IWRTE* impact of war-related traumatic events, *LEC-5* Life Event Checklist for DSM-5, *PCL-5* Posttraumatic Stress Disorder Checklist, *PHQ-9* Patient Health Questionnaire, 9-item module, *GAD-7* Generalized Anxiety Disorder Scale, *SSS-8* Somatic Symptoms Scale, *TRGI* Trauma-related Guilt Inventory, *SVQ* Shame Variability Questionnaire, *CHQ* Child Health Questionnaire, *WAI* Weinberger Adjustment Inventory, *YSR* Youth Self-Report

**Table 2:** Summary of findings of the included studies

Study	Risk factor	Protective factor	No effect
**Age**			
Bean et al. (2007a)	Older		
Bean et al. (2007b)^a^	Older		
Bronstein et al. (2012)			Age
Bronstein et al. (2013)	Older		
Derluyn et al. (2009)			Age
Hodes et al. (2008)	Older		
Hollins et al. (2007)	Older		
Jakobsen et al. (2017)^a^			Age
Jensen et al. (2014) (l)			Age
Jensen et al. (2015)			Age
Keles et al. (2015)			Age
Müller et al. (2019)			Age
Oppedal and Isdoe (2015)	Older		
Porte and Torney-Purta (1987)			Age
Rücker et al. (2017)	Younger		
Smid et al. (2011)^a^	Older		
Sourander (1998)	Younger		
**Gender**			
Bean et al. (2007a)	Female		
Bean et al. (2007b)^a^	Female		
Derluyn et al. (2009)	Female		
Hodes et al. (2008)	Female		
Jensen et al. (2014)^a^			Gender
Jensen et al. (2015)			Gender
Keles et al. (2015)	Female		
Keles et al. (2018)^a^	Female	Female	
Oppedal and Isdoe (2015)	Female/Male		
Reijneveld et al. (2005)	Female		
Smid et al. (2011)			Gender
Sourander (1998)			Gender
Vervliet et al. (2014)^a^	Female		
Völkl-Kernstock et al. (2014)	Female		
**Origin**			
Bean et al. (2007b)^a^	Ethiopia/Eritrea		Country of origin
Bronstein et al. (2012)			Language of origin
Bronstein et al. (2013)			Language of origin
Hodes et al. (2008)	Male african/Middle east		Country of origin
Jakobsen et al. (2017)^a^			Country of origin
Jensen et al. (2015)			Country of origin
Müller et al. (2019)			Country of origin
Sourander (1998)			Country of origin
**Stressful life events**			
Bean et al. (2007a)	Number of SLE		
Bean et al. (2007b)^a^	Number of SLE		
Bronstein et al. (2012)	Number of SLE		
Bronstein et al. (2013)	Number of SLE		
Derluyn et al. (2009)	Number of SLE		
Entholt et al. (2018)	Number of SLE Having age disputed		
Geltman et al. (2005)	Physical injury		Witnessing violence
Hodes et al. (2008)	Number of SLE		
Jakobsen et al. (2017)^a^	Parents deceased		Number of SLE
Jensen et al. (2014)^a^	Number of post-migration SLE		
Jensen et al. (2015)	Number of SLE		
Keles et al. (2015)	Exposure to war traumata Cultural and daily hassles		
Keles et al. (2018)^a^	Number of SLE Cultural and daily hassles		
Müller-Bamouth et al. (2016)	Experienced organized violence Experienced family violence		
Müller et al. (2019)	Number of SLE		Discrimination
Oppedal and Isdoe (2015)	Discrimination		
Rücker et al. (2017)			Duration of flight
Smid et al. (2011)^a^	Number of SLE		
Stotz et al. (2015)	Number of SLE		
Vervliet et al. (2014)^a^	Number of SLE Cultural daily hassles		
Völkl-Kernstock et al. (2014)	Number of SLE		
**Individual competences**			
Geltman et al. (2005)			Cultural adjustment
Huemer et al. (2013)		Restraint and Defensiveness	
Keles et al. (2018)^a^		Cultural competences	
Müller et al. (2019)		Everyday resources Language skills	
Oppedal and Isdoe (2015)		Cultural competences	
**Time spent in host country**			
Bean et al. (2007a)			Time spent in host country
Bean et al. (2007b)^a^	Increasing time		
Bronstein et al. (2012)			Time spent in host country
Bronstein et al. (2013)	Increasing time		Time spent in host country
Derluyn et al. (2009)			Time spent in host country
Geltman et al. (2005)			Time spent in host country
Hodes et al. (2008)			Time spent in host country
Jakobsen et al. (2017)^a^			Time spent in host country
Jensen et al. (2014)^a^			Time spent in host country
Jensen et al. (2015)			Time spent in host country
Keles et al. (2015)			Time spent in host country
Keles et al. (2018)^a^		Increasing time	
Oppedal and Isdoe (2015)		Increasing time	
Porte and Torney-Purta (1987)			Time spent in host country
Rücker et al. (2017)			Time spent in host country
Vervliet et al. (2014)^a^			Time spent in host country
**Accommodation**			
Bean et al. (2007b)^a^	Low support accommodation Change of residence	Foster care	
Bronstein et al. (2012)	Low support accommodation	Foster care	Change of residence
Bronstein et al. (2013)		Foster care	Change of residence
Geltman et al. (2005)	Without other UMR		Urban environment
Hodes et al. (2008)	Low support accommodation	Foster care Living with family member	
Hollins et al. (2007)	Low support accommodation		
Jakobsen et al. (2017)^a^	Low support accommodation		
Porte and Torney-Purta (1987)		Ethnic foster family Living with family member	
Reijneveld et al. (2005)	High restricted reception setting	Low restricted reception setting	
**Status of residence**			
Bean et al. (2007b)^a^	No permanent residence status		
Jakobsen et al. (2017)^a^	Refusal of asylum claims		Temporary residence status
Rücker et al. (2017)			Residential status
Smid et al. (2011)^a^			Residential status
**Social support**			
Bean et al. (2007b)^a^	Mental healthcare services	Mental healthcare services	
Geltman et al. (2005)	Feeling alone /isolated	Social interaction	
Müller et al. (2019)		Social support	
Oppedal and Isdoe (2015)		Social support	
Porte and Torney-Purta (1987)		Social support	
Sierau et al. (2019)		Social support	
**Education**			
Geltman et al. (2005)		Safe school environment	
Jakobsen et al. (2017)^a^			Educational background
Müller et al. (2019)			Educational background
Rücker et al. (2017)	Having a school diploma		Educational background
Smid et al. (2011)^a^	Low education level		
**Family**			
Bean et al. (2007b)^a^		Family member in host country	
Hollins et al. (2007)	No contact to family		
Müller et al. (2019)			Family support (moderator)
Oppedal and Isdoe (2015)		Family contact	
Rücker et al. (2017)			Financial status of family
Sierau et al. (2019)	No contact to family	Family support	

^a^Longitudinal study design

### Age

Age does not seem to be a very distinct predictor of psychological distress for UMR. Out of 17 studies, nine could find a link between age and psychological parameters [5, 6, 9, 16, 24–28]. Five cross-sectional and two longitudinal studies reported that an increase in age was related to an increase in psychological distress [5, 6, 16, 24–26, 28].Two cross-sectional studies with a disproportionately high number of male UMR in their study sample stated that younger UMR tend to develop more mental health problems than older UMR [9, 27]. One of these two studies reports the effect only for externalizing outcome measures [27]. Six cross-sectional and two longitudinal studies could not link age to psychological distress [7, 29–35].

### Gender

In contrast to age, the results of the studies investigating gender as a predictive factor are more consistent. Seven cross-sectional studies reported female gender represents a risk factor for different internalizing psychological distress outcomes in UMR [5–7, 25, 30, 36, 37]. One of these cross-sectional studies reported a significant correlation between female gender and depressive symptoms, but, at the same time, between male gender and symptoms of PTSD [6]. A longitudinal study reported a link between female gender and the development of intrusive posttraumatic stress symptoms over time [38]. Another longitudinal study reports only small effect sizes for the connection between female gender and internalizing and posttraumatic stress symptoms [16]. One longitudinal study found female UMR to be at a higher risk for developing mental health problems, but at the same time to be more resilient compared to male UMR [8]. The remaining one longitudinal and three cross-sectional studies did not report any predictive value for gender on psychological distress [27, 28, 32, 33].

### Origin

Eight studies investigated the influence of the country or language of origin on psychological distress of UMR [16, 24, 25, 27, 29, 31, 32, 34]. Three cross-sectional studies and one longitudinal study did not find any impact of country of origin on distress [27, 31, 32, 34]. Two cross-sectional articles which are based on the same examined sample of UMR from Afghanistan reported no difference regarding psychological distress between Dari- or Pashto-speaking UMR [24, 29]. In one large longitudinal study, being migrated from Ethiopia or Eritrea was a predictor for developing externalizing behavior problems over time. Whereas for the development of internalizing problems the country of origin showed no predictive value [16]. One cross-sectional study reported that having migrated from middle-eastern countries or from African countries (only male UMR) increases the risk of developing depressive symptoms in comparison to UMR immigrating from Europe, Asia or South America [25]. However, for symptoms of PTSD, this effect could not be shown in this study.

### Stressful life events

In the studies included, SLE are the most frequently investigated and confirmed risk factors. Overall, 21 of the 27 studies investigated the experience of SLE on mental health in some way. A total of ten cross-sectional and five longitudinal studies found that the more SLE (number of* SLE*) were experienced by UMR the higher their psychological distress level was [5, 8, 16, 24, 25, 28–30, 32–34, 36, 38–40]. One longitudinal study with an all-male study population could not replicate this general finding [31]. However this study reports a statistically significant negative impact of deceased parents on PTSD-related symptoms. Furthermore, some other specific SLE were investigated separately. For example, the exposure to war traumata was found to be highly predictable for future psychological distress of UMR in a large and representative cross-sectional study [7]. In a cross-sectional study with a comparatively small sample size and an all-male study population the experience of organized violence and family violence was reported to be a predictor for increasing symptoms of PTSD [41]. In a cross-sectional study with only Sudanese UMR the experience of physical injury or assault predicted a negative mental health outcome [42]. However, witnessing violence had no predictive value on mental health outcomes in this study. A small cross-sectional study with an almost all male study population investigated the influence of the factor duration of flight. Although this factor is not an SLE by itself, it corresponds most closely to this category. The study reported no significant association between duration of flight and psychological distress [9]. Three longitudinal studies and one cross-sectional studies also investigated post-migratory SLE such as cultural daily hassles [7, 8, 33, 38]. These factors might not be as invasive as, for example, the exposure to war traumata, but they had a comparably strong negative impact on the mental wellbeing of UMR. A small but representative cross-sectional study reported a negative effect of having age disputed by authorities on symptoms of depression and PTSD (measured by impartial opinion of clinicians) [39]. Additionally, one large cross-sectional study reported perceived discrimination to be highly associated with depressive symptoms [6]. However, a smaller cross-sectional study with a less representative study population did not replicate this finding in their regression analysis [34].

### Individual competences

While most studies focused on demographic variables as predictors, there were only five studies in this review that investigated individual competences in relation to psychological distress. Four studies (one longitudinal) report that specific competences can be protective regarding mental health [6, 8, 34, 43]. Especially UMR with a high level of cultural competences seem to be more resilient. Cultural competences were defined as knowledge and skills about verbal and non-verbal communication and interpersonal behavior patterns to develop a sense of belongingness both within the heritage and host culture [6]. The protective effect of cultural competences on mental health was reported in two large and representative studies (one longitudinal, one cross-sectional) [6, 8]. Cultural adjustment, on the other hand, did not have a beneficial effect on mental health in cross-sectional study with Sudanese UMR. In this study, cultural adjustment was defined as how comfortable you feel with the host society and culture [42]. A cross-sectional study with almost only male participants reports that UMR, who had high levels of everyday resources (e.g., practicing sports) showed lower levels of externalizing behavior problems [34]. Further, they report that high language skills predicted lower levels of symptoms of depression and PTSD, as it helps individuals to communicate with others and to express their feelings and needs. Another rather small cross-sectional study examined competences with regard to personality traits in African UMR [43]. They report that being a more controlled and defensive personality type in dealing with psychological distress helped them to keep these symptoms in check at first. On the long run, however, these personality traits did not protect UMR from psychological distress.

### Time spent in the host country

The amount of time spent in the host country seems to have no additional predictive value regarding psychological distress. Only 4 out of 16 investigating studies reported a predictive effect of time since arrival on mental health outcomes [6, 8, 16, 24]. One large and representative longitudinal study describes a negative effect of an increased time spent in the host country on externalizing behavior problems [16]. This effect on externalizing symptoms was replicated for male Afghan UMR by a cross-sectional study [24]. On the other hand, one longitudinal and one cross-sectional study, reported a reduction of internalizing problems with an increased time spent in the host country [6, 8].

### Accommodation

The predictor accommodation was investigated in more detail compared to some other predictors of this review. One longitudinal and one cross-sectional study reported a protective effect of living in a foster care accommodation on symptoms of depression and PTSD in UMR [16, 25]. This effect was replicated for symptoms of PTSD and general mental health problems in two studies which were based on a male Afghan study population [24, 29]. The oldest cross-sectional study of this review with an only Indochinese study sample reported a protective effect on symptoms of depression only for ethnic foster families [35]. Another cross-sectional study finds Sudanese UMR at a higher risk of developing psychological distress if they are placed in an accommodation without other UMR [42]. Two cross-sectional studies reported a protective effect on mental health if UMR were able to live together with one of their family members [25, 35]. Two longitudinal and three cross-sectional studies report that UMR which were placed in big refugee camps, reception centers or other kinds of more low support accommodations are at a higher risk of developing psychological distress [16, 25, 26, 29, 31]. A higher number of changes of accommodations was found to have a negative impact in one large longitudinal study [16], whereas two cross-sectional studies could not replicate this result on a male Afghan study population [24, 29]. One cross-sectional study reported that Sudanese UMR which were placed in either an urban or more rural environment did not differ in terms of their psychological wellbeing [42]. A more influential factor on psychological distress seems to be the degree of restrictions within a reception center. One cross-sectional study, investigating primarily African UMR, reported that a restricted reception setting puts the mental health of UMR at a greater risk than a more autonomic reception setting [37].

### Status of residence

Although it seems plausible that the status of residence might have an impact on mental health in terms of increasing fears of deportation, it has not been evaluated as much as other potential predictors. While one large longitudinal study with a representative study population reported that having no permanent status of residence can be considered as a risk factor of internalizing mental health problems in UMR [16], a comparable longitudinal study did not report an impact of residential status on mental health outcomes [28]. A small cross-sectional study on a mainly male UMR study sample also did not report an effect of residential status on psychological distress [9]. Another longitudinal study investigated the influence of the residential status on only male UMR in more detail [31]. A temporary residence status was not predictive for mental health outcomes but the refusal of asylum claims was found to be a risk factor for psychological distress.

### Social support

According to four cross-sectional studies with various study populations, there is strong evidence that receiving social support is a reliable protective factor for different mental health outcomes in UMR [6, 34, 35, 44]. Further, receiving social support can also have a positive impact on the protective factor cultural competences [6]. These findings are supported by a study reporting that feeling alone and isolated led to a deterioration of mental health in Sudanese UMR and that being in some kind of regular social interaction had a positive influence on their mental health [42]. However, it must be taken into account that all these studies had a cross-sectional design and causality is not statistically ensured. A large longitudinal study from the Netherlands investigated the influence of mental healthcare services on different psychological outcomes [16]. UMR who had received any kind of mental healthcare services reported significantly less internalizing symptoms and less symptoms of PTSD at T2. But, at the same time, externalizing behavior problems increased significantly in UMR who made use of mental healthcare services.

### Education

The impact of the educational background on psychological distress of UMR has been investigated in four studies [9, 28, 31, 34]. One longitudinal (only male UMR) and two cross-sectional studies (almost all male UMR) showed no significant impact of the length or the level of former education on psychological distress. However, one of two cross-sectional studies mentioned a small but statistically significant connection between increased emotional distress and having a school diploma [9]. In contrast, a large longitudinal study investigating the influence of educational background on late-onset PTSD reported a low level of education to be a risk factor [28]. In addition, the school environment in the host country can also have a predictive value on psychological distress. A cross-sectional study reported that a safe school environment had a protective effect on internalizing mental health outcomes within Sudanese UMR [42].

### Family

There are various factors that can be considered when investigating the influence of family on psychological distress in UMR. As mentioned earlier, the loss of a family member or the experience of violence within the family can be considered as a great burden for the mental health of UMR [31, 41]. But, even after arrival in the host country, family can play an important role in the development of the mental health problems. Two cross-sectional studies reported various positive effects of family contact and family support [6, 44]. For example, the larger and more representative study reported a protective impact of family contact on symptoms of depression. Further they report an increase in cultural competences when having more family contact [6]. The other, more recent study with only male UMR finds family support to be a protective factor for internalizing symptoms [44]. Further this study reports family support to be an attenuating moderator for the negative impact of SLE on mental health. In accordance with these results, a large longitudinal study reported a protective impact of having a family member in the host country on internalizing psychological symptoms, as well as on symptoms of PTSD with overall medium effect sizes [16]. Although one cross-sectional study with almost only male UMR did not confirm family support as a direct protective factor for mental health outcomes, it reported family support to be protective in terms of the number of SLE experienced by UMR [34]. The importance of family contact becomes even more crucial if you take a look at the mental health outcomes when family contact is lacking. Having no contact with the family was significantly correlated with an increased risk of psychological distress in two cross-sectional studies [26, 44]. In a small cross-sectional study (almost all male UMR) the former financial status of the family of UMR, however, seemed to have no additional predictive value regarding an emotional distress outcome [9].

### Summary of results

In this review, we found the number of SLE to be the most evaluated and verified risk factor for mental health problems in UMR. A predictive effect of the number SLE was found in more than half of the investigated studies regardless of the outcome measures, study designs or sample characteristics. These results are consistent with similar findings for minor refugees in general [18, 20]. In addition to the number of SLE, this review identifies female gender and low support accommodations to be well-evaluated risk factors of psychological distress. On the other hand, social support, high support living arrangement, contact with family members and high levels of cultural competences within UMR are well-confirmed protective factors which can strengthen the mental health of UMR. Further, there seems to be a double-sided effect of the supportive and social predictors, which increases their importance substantially. Given the results of the investigated studies, factors like age, origin, residential status, time spent in host country and educational background cannot be considered as reliable predictive factors for mental health problems in UMR.

## Discussion

This is the first review to have systematically investigated predictive factors of the mental health in UMR. In summary, it can be stated that there are multiple risk and protective factors of psychological distress in UMR. These factors vary strongly in their degree of verification. The factors were assessed on wide-ranging levels from pre-migration to post-migration factors and from more individual to more societal factors. This review summarized and interpreted the most important and verified findings to provide a comprehensive overview of the risk and protective factors of the mental health of UMR. The main results of this review are supported by very similar findings of a recent longitudinal study investigating pre- and post-flight predictors [45].

Overall, risk factors were more often investigated than protective factors. This complicates the provision of supportive practical help by health care systems. Most of the evaluated risk factors lie in the past and cannot be changed by authorities in charge anymore. It is very important for future studies to gain more knowledge about preventive and changeable factors to develop more effective interventions. As mentioned earlier, many studies reported a remarkable resilience in UMR considering these stressful circumstances. They highlighted the importance of future investigation concerning aspects of resilience in UMR [4, 9]. One general problem concerning the investigation of risk and protective factors is that the differentiation between these factors is not always clear. Sometimes they represent two sides of the same coin. For example, if female gender is supposed to be a risk factor for psychological distress, male gender could be seen as a protective factor as well. The same applies for many other factors such as age and family contact. The categorization of these factors sometimes appears to be arbitrary and vague. We advise future investigators to carefully justify their definition of risk or protective factors. Another way to handle this issue would be to no longer label factors as risk or protective factors but to label them as predictive factors and to describe the direction of the influence accordingly [45].

As seen in the results, it is difficult to link age directly to psychological distress. The studies reporting a predictive effect of age on mental distress mainly found an older age to be associated with increased mental health problems. This effect might also be explained by various potentially mediating variables. For example, older UMR have, in general, experienced more SLE [6, 28, 30, 34] and they often have to live in less supportive living arrangements [25] compared to younger UMR. Furthermore, their residential status is more likely to be critically reviewed at the age of 18. This may increase their fear of being deported and, in turn, might lead to additional mental health problems. On the other hand, an increasing likelihood of social support by health authorities might attenuate these risk factors. The confoundation by different mediators can also find application for the factor time spent in host country. Many new daily stressors can in- or decrease over time, such as being repeatedly moved, experiencing discrimination or missing the family [38]. At the same time UMR can extend their social contacts over time, get psychological treatment and develop cultural competences. Thus, the investigation of the time spent in host country as a predictive factor seems impractical for future research, especially when applying a cross-sectional study design. According to the results of the investigated studies, female gender can be considered a reliable risk factor. Gender being a risk factor has been found in the trauma literature as well and may also be indicative of an increased experience of SLE (like unreported sexual abuse) [46]. These results are in line with the findings for young refugees in general [18]. However, this gender effect might also partially be influenced by unbalanced outcome measures of the included studies. Most studies in this review assessed primarily internalizing symptoms, which are known to be more common in females [47, 48]. Further studies investigating more differentiated outcome measures (especially with regard to externalizing symptoms) are needed before deriving global conclusions of the gender-specific findings in this review. Furthermore, most of the study populations consisted of primarily male UMR. This gender gap in the study populations was probably due to the fact that mostly male refugees flee from their countries of origin unaccompanied [1]. Unfortunately, this complicates an adequate exploration of the risk factor gender.

### Limitations

The studies included in this review were of varying designs and sample sizes. Limitations of the work therefore include heterogeneous study designs and outcomes, and, in most cases, the lack of reported effect sizes according to Cohen [49], which limits the ability to draw definitive conclusions. Although a quantitative synthesis of the studies in terms of a meta-analysis may seem desirable for our research question, it seemed unreasonable for us to do so, given the large heterogeneity regarding the outcome measures of the included studies. We therefore decided to conduct a qualitative aggregation of the results. A major limitation of this review is the restricted reporting of the different outcome measures for each predictive factor. A more detailed reporting would have helped to derive more reliable and precise implications for different psychiatric disorders. However, to keep this review clear and legible, we decided to subsume the different outcome measures and, as a result, derive more general statements about the mental health of UMR. Furthermore, the studies were mainly carried out in high-income countries, which limits the generalizability of findings to UMR who immigrated to low- and middle-income countries [20]. Since most UMR immigrate to low- and middle-income countries it is crucial to support corresponding research in these countries [1]. Except for one small cross-sectional study, the assessment of predictors lacked the investigation of personality traits [43]. Considering the remarkable rates of resilience within UMR, links to characteristics within the individual should be researched more intensively. Moreover, risks and protective factors cannot be simply added up [50]. It is important for future research to analyze the inter-related pathways that lead to the outcome measures, especially whether and how factors mediate or moderate the effects of stressors. Further, the dominance of cross-sectional approaches is a major limitation in the identification of factors that affect the mental health of UMR at different stages of their life. In this review, every general predictor was assessed by at least one longitudinal study. The causality of the predictive factors identified in studies with a cross-sectional design should be critically questioned. Therefore, we referred to the different study designs in the reporting of the results. Long-term outcomes should be given more consideration in future studies, since they offer additional information on the development and the impact of predictive factors.

## Conclusion

The results revealed that the wellbeing of UMR is strongly influenced by the experience of SLE. It is important to notice that the examined SLE were not limited to pre- or peri-migration phases but to post-migrant SLE as well (e.g., perceived discrimination). After experiencing multiple SLE in the past it can be very devastating to experience further SLE and a lack of safety after migration. The results concerning the remarkable influence of SLE indicate that governmental health and immigration services should put increased attention into capturing information about former SLE via an early screening and further preventing new SLE after resettlement. One preventive approach to reduce post-migrant SLE could be to support ethnic diversity and respectful interactions in communities and schools. For clinicians these results implicate to be aware of the high level of past and present SLE of UMR and to compile effective coping strategies in dealing with them.

The results further strongly implicate that a lack of support and perceived social isolation puts UMR at a great risk of developing mental health problems whereas social support and in particular family contact positively affects their mental health. This influence has been confirmed in multiple ways in the studies identified. Health authorities should acknowledge these findings and increase their efforts to strengthen social participation of UMR. Especially the preventive effect of family contact seems to be promising. Since most UMR are not surrounded by their family after resettlement, it is of major importance to help UMR to get in contact with their families, whether through family reunification or by enhancing media-supported contact. Thereby, they can be reassured of the wellbeing of their family in conflict areas and have the opportunity to talk about personal problems with a trusted family member. Although the promotion of family contact in UMR seems to be very helpful in terms of mental health of UMR, only a few studies have investigated this influence so far.

The types of accommodation seem to have a great influence on the wellbeing of UMR. Living in foster care can be considered a reliable protective factor, presumably due to the higher level of social support, safety and stability within this form of accommodation. Furthermore, UMR living in low support accommodation are more likely to become socially isolated and to report increased mental health problems. These findings endorse the protective impact of social support in general and they represent a concrete example of how to implicate this promising factor in terms of living arrangements. The promotion of high supportive living arrangement (e.g., foster care) by public and governmental funding seems to be a very helpful approach in preventing UMR from developing mental health problems.

The protective effect of cultural competences and the lacking influence of cultural adjustment supports recent theories about acculturation, stating that it is more helpful to be aware of your own culture and the culture of the host country than trying to adjust to a new culture [51]. These findings indicate that professionals and health administrators should not only focus on developing better coping skills regarding traumatic experiences but to teach and establish cultural competences at an early stage to strengthen the resilience of UMR.

To sum up, this review highlights the negative psychological impact of stressful life events on UMR and indicates that different kinds of social support might help fostering their mental health. Special attention should be paid to the promotion of cultural competences and the early access to supportive and safe housing. This could help to minimize mental health problems of this highly vulnerable group.

## Electronic supplementary material

Below is the link to the electronic supplementary material.

Supplementary file1 (DOCX 24 KB)

Supplementary file2 (DOCX 30 KB)
