# Supplementary material for: A systematic review of risk and protective factors of mental health in unaccompanied minor refugees
Source: Eur Child Adolesc Psychiatry. 2020 Nov 9;31(8):1–15. doi: 10.1007/s00787-020-01678-2 (PMC9343263; doi:10.1007/s00787-020-01678-2)
Supplement: Supplementary file 1 — Supplementary file1 (DOCX 24 KB) [file 787_2020_1678_MOESM1_ESM.docx]

# **Appendix A:** *Search terms by AND-categories and languages*

| **language** | **predictive factors** | **sample** | **accompaniment** | **refugee status** |
| --- | --- | --- | --- | --- |
| German | Stressor* | Minderjährig* | Unbegleitet* | Flüchtling* |
|  | Resilien* | Kind* |  | Geflüchtet* |
|  | Schutzfaktor* | Jugendliche* |  | Ausländer* |
|  | Risikofaktor* |  |  | Migrant* |
|  | Schutz* |  |  | Asylsuchend* |
|  | Risik* |  |  | Asylbewerber* |
|  | Ressource* |  |  | Fluchterfahrung* |
|  |  |  |  | Fluchthintergrund* |
| English | Resilien* | Minor* | Unaccompanied | Refugee* |
|  | Protective | Youth |  | Immigrant* |
|  | Resource* | Adolescent* |  | Asylum-seek* |
|  | Risk* | Teenage* |  | Displaced |
|  | Stressor* | Child* |  | Migrant* |
|  | Protector* |  |  |  |
| French | Facteur* de risque | Mineur* | Isolé* | Étrange* |
|  | Risque* | Enfant* | Non accompagné* | Fugiti* |
|  | Facteur* de protection | Adolescent* |  | Réfugié* |
|  | Protecteur* | Jeune* |  | Demandeu* d‘asile |
|  | Résilien* |  |  | Émigré* |
|  | Stress* |  |  | Immigré* |
| Dutch | Stressor* | Minderjarig* | Alleenstaand* | vluchteling* |
|  | Beschermingsfactor* | Kind* |  | gevlucht |
|  | Risicofactor* | Jongere* |  | buitenlander* |
|  | bescherming | onvolwassen |  | allochtoon* |
|  | Risico* | Jeugdige* |  | migrant* |
|  | hulpbron*/ ressource* | Adolescent* |  | asielzoek* |
|  |  |  |  | asielaanvrager* |
|  |  |  |  | Vluchtervaring* |
|  |  |  |  | Vluchtachtergrond/ vluchtoorzaak |

*Table 2. Results of literature search from 04/2018 – 03/2019*

|  |  | | |  | | | |  |  |  | |  |  |
| --- | --- | --- | --- | --- | --- | --- | --- | --- | --- | --- | --- | --- | --- |
| **Database** | **Language** |  | **new results** | | | **duplicates removed** | **records screened** | | | | **full-texts assesed** | | **new studies included** |
| **Pubmed** | Englisch | | | 26 | 161 | 97 | 97 | | | | 14 | | 3 |
|  | Deutsch | | | 0 |  |  |  |  |  |  |  |  |  |
|  | Französisch | | | 0 |  |  |  |  |  |  |  |  |  |
|  | Holländisch | | | 0 |  |  |  |  |  |  |  |  |  |
| **PsycINFO** | Englisch | | | 17 |  |  |  |  |  |  |  |  |  |
|  | Deutsch | | | 2 |  |  |  |  |  |  |  |  |  |
|  | Französisch | | | 0 |  |  |  |  |  |  |  |  |  |
|  | Holländisch | | | 0 |  |  |  |  |  |  |  |  |  |
| **PSYNDEX** | Englisch | | | 5 |  |  |  |  |  |  |  |  |  |
|  | Deutsch | | | 6 |  |  |  |  |  |  |  |  |  |
|  | Französisch | | | 0 |  |  |  |  |  |  |  |  |  |
|  | Holländisch | | | 0 |  |  |  |  |  |  |  |  |  |
| **Web of Science** | Englisch | | | 61 |  |  |  |  |  |  |  |  |  |
|  | Deutsch | | | 0 |  |  |  |  |  |  |  |  |  |
|  | Französisch | | | 0 |  |  |  |  |  |  |  |  |  |
|  | Holländisch | | | 0 |  |  |  |  |  |  |  |  |  |
| **Google Scholar** | Englisch | | | 13 |  |  |  |  |  |  |  |  |  |
|  | Deutsch | | | 13 |  |  |  |  |  |  |  |  |  |
|  | Französisch | | | 0 |  |  |  |  |  |  |  |  |  |
|  | Holländisch | | | 0 |  |  |  |  |  |  |  |  |  |
| **ERIC** | Englisch | | | 0 |  |  |  |  |  |  |  |  |  |
|  | Deutsch | | | 0 |  |  |  |  |  |  |  |  |  |
|  | Französisch | | | 0 |  |  |  |  |  |  |  |  |  |
|  | Holländisch | | | 0 |  |  |  |  |  |  |  |  |  |
| **Cochrane Libary** | Englisch | | | 0 |  |  |  |  |  |  |  |  |  |
|  | Deutsch | | | 0 |  |  |  |  |  |  |  |  |  |
|  | Französisch | | | 0 |  |  |  |  |  |  |  |  |  |
|  | Holländisch | | | 0 |  |  |  |  |  |  |  |  |  |
| **PubPsych** | Englisch | | | 13 |  |  |  |  |  |  |  |  |  |
|  | Deutsch | | | 5 |  |  |  |  |  |  |  |  |  |
|  | Französisch | | | 0 |  |  |  |  |  |  |  |  |  |
|  | Holländisch | | | 0 |  |  |  |  |  |  |  |  |  |
| **other sources** |  | | |  |  |  |  | | | | |  |  |
|  |  | | |  |  |  |  | | | | |  |  |
|  |  | | |  |  |  |  | | | | | 12 | 3 |
|  |  | | |  |  |  |  | | | | |  |  |
| **SUMME** |  | | | **161** |  |  |  | | | | |  | 6 |
