# Supplementary material for: A systematic review of risk and protective factors of mental health in unaccompanied minor refugees
Source: Eur Child Adolesc Psychiatry. 2020 Nov 9;31(8):1–15. doi: 10.1007/s00787-020-01678-2 (PMC9343263; doi:10.1007/s00787-020-01678-2)
Supplement: Supplementary file 2 — Supplementary file2 (DOCX 30 KB) [file 787_2020_1678_MOESM2_ESM.docx]

|  |  |  | Bean et al., 2007b | Bean et al., 2007a | Bronstein et al., 2013 | Hodes et al., 2008 | Hollins et al., 2007 | Rücker et al. ,2017 | Smid et al., 2011 | Sourander, 1998 | Völkl-Kernstock et al., 2014 |
| --- | --- | --- | --- | --- | --- | --- | --- | --- | --- | --- | --- |
| Study design |  |  | cross | long | cross | cross | cross | cross | long | cross | cross |
| AXIS Quality |  |  | 18/20 | 18/20 | 19/20 | 17/20 | 16/20 | 14/20 | 19/20 | 13/20 | 16/20 |
| Risk of bias |  |  | low | low | low | medium | medium | medium | low | high | medium |
| Introduction | 1 | Clear aims? | yes | yes | yes | yes | yes | yes | yes | yes | yes |
| Methods | 2 | Appropriate study design? | yes | yes | yes | yes | yes | yes | yes | yes | yes |
|  | 3 | Justified sample size? | no | no | no | no | no | no | no | no | no |
|  | 4 | Clearly defined population? | yes | yes | yes | yes | yes | yes | yes | yes | yes |
|  | 5 | Appropriate sample population? | yes | yes | yes | yes | yes | yes | yes | yes | yes |
|  | 6 | Process selects representative sample? | yes | yes | yes | yes | yes | yes | yes | yes | yes |
|  | 7 | Addresses and categorises non-responders? | yes | yes | yes | yes | no | no | yes | no | yes |
|  | 8 | Appropriate outcome variables? | yes | yes | yes | yes | yes | yes | yes | no | yes |
|  | 9 | Valid instruments to measure outcomes? | yes | yes | yes | yes | yes | yes | yes | yes | yes |
|  | 10 | Statistical significance clear? | yes | yes | yes | yes | yes | yes | yes | yes | yes |
|  | 11 | Methods described enable to be replicated? | yes | yes | yes | yes | yes | yes | yes | yes | yes |
| Results | 12 | Basic data described adequately? | yes | yes | yes | yes | yes | yes | yes | yes | yes |
|  | 13 | Non-response bias concern? | no | no | no | yes | yes | yes | no | yes | yes |
|  | 14 | Non-responders described? | yes | yes | yes | no | no | no | yes | no | no |
|  | 15 | Results internally consistent? | yes | yes | yes | yes | yes | yes | yes | yes | no |
|  | 16 | Results presented for all method analyses? | yes | yes | yes | yes | yes | yes | yes | yes | yes |
| Discussion | 17 | Conclusion justified by results? | yes | yes | yes | yes | yes | yes | yes | yes | yes |
|  | 18 | Limitations discussed? | yes | yes | yes | yes | yes | yes | yes | no | yes |
| Other | 19 | Funding or conflict of interest concern? | - | - | yes/- | no | no | - | yes/- | yes/- | no |
|  | 20 | Ethical approval or consent obtained? | yes | yes | yes | yes | yes | no | yes | no | yes |
| Comments |  |  | 3: not discussed  19: no statement | 3: not discussed  19: no statement | 3: not discussed  13: 32% non-participants  14: no significant differences between participants and non-participants  19: funded but no apperent conflict of interest | 3: not discussed  13: response rate 57%, unclear flow of participants, only N=70  14: insufficient information  18: only brief description | 3: not discussed  7: mentioned, but not discussed  13: 50% response rate  14: briefly described but not suffiently discussed | 3: not discussed  7: not discussed  13: no response rate given  14: not discussed  19: no statement  20: no statement  + questionable age classification | 3: not discussed  14: reasons mentioned, detailed characteristica in different paper  19: funded but no apperent conflict of interest | 3: not discussed  7: not discussed  8: changed outcome + outcome measure is not culturally apropriate  13: no response rate given  14: not discussed  18: not discussed  19: funded but no apperent conflict of interest  20: no statement | 3: not discussed  13: response rate = 64%, small N  14: not discussed  15: N=41 vs. N= 40 in the results without further explanation |

*Comments addressing where studies deviated from quality appraisal tool. Numbers in boxes correspond to question numbers.*

|  |  |  | Vervliet et al., 2014 | Reijneveld et al. ,2005 | Keles et al., 2015 | Derluyn et al., 2009 | Keles et al., 2018 | Jensen et al., 2015 | Jensen et al., 2014 | Bronstein et al., 2012 | Jakobsen et al., 2017 |
| --- | --- | --- | --- | --- | --- | --- | --- | --- | --- | --- | --- |
| Study design |  |  | Long | cross | cross | cross | long | cross | long | cross | long |
| AXIS Quality |  |  | 18/20 | 19/20 | 19/20 | 19/20 | 19/20 | 16/20 | 19/20 | 19/20 | 19/20 |
| Risk of Bias |  |  | low | low | low | low | low | medium | low | low | low |
| Introduction | 1 | Clear aims? | yes | yes | yes | yes | yes | yes | yes | yes | yes |
| Methods | 2 | Appropriate study design? | yes | yes | yes | yes | yes | yes | yes | yes | yes |
|  | 3 | Justified sample size? | no | no | no | no | no | no | no | no | no |
|  | 4 | Clearly defined population? | yes | yes | yes | yes | yes | yes | yes | yes | yes |
|  | 5 | Appropriate sample population? | yes | yes | yes | yes | yes | yes | yes | yes | yes |
|  | 6 | Process selects representative sample? | yes | yes | yes | yes | yes | yes | yes | yes | yes |
|  | 7 | Addresses and categorises non-responders? | yes | yes | yes | yes | yes | no | yes | yes | yes |
|  | 8 | Appropriate outcome variables? | yes | yes | yes | yes | yes | yes | yes | yes | yes |
|  | 9 | Valid instruments to measure outcomes? | yes | yes | yes | yes | yes | yes | yes | yes | yes |
|  | 10 | Statistical significance clear? | yes | yes | yes | yes | yes | yes | yes | yes | yes |
|  | 11 | Methods described enable to be replicated? | yes | yes | yes | yes | yes | yes | yes | yes | yes |
| Results | 12 | Basic data described adequately? | yes | yes | yes | yes | yes | yes | yes | yes | yes |
|  | 13 | Non-response bias concern? | no | no | no | no | no | yes | no | no | no |
|  | 14 | Non-responders described? | no | yes | yes | no | yes | no | yes | yes | yes |
|  | 15 | Results internally consistent? | yes | yes | yes | yes | yes | yes | yes | yes | yes |
|  | 16 | Results presented for all method analyses? | yes | yes | yes | yes | yes | yes | yes | yes | yes |
| Discussion | 17 | Conclusion justified by results? | yes | yes | yes | yes | yes | yes | yes | yes | yes |
|  | 18 | Limitations discussed? | yes | yes | yes | yes | yes | yes | yes | yes | yes |
| Other | 19 | Funding or conflict of interest concern? | no | yes/no | no | yes/no | no | yes/no | no | yes/no | no |
|  | 20 | Ethical approval or consent obtained? | yes | yes | yes | yes | yes | yes | yes | yes | yes |
| Comments |  |  | 3: not discussed  14: only reasons for drop-out mentioned, no chracteristica | 3: not discussed  10: only p-values mentioned -significance level not described  19: funding yes but no apprent conflict of interest | 3: not discussed  13: response rate 78%  14: non-participants did not differ from participants | 3: not discussed  14: only one non-responder  19: funding yes but no apparent conflict of interest | 3: not discussed  13: response rate at T2 63%  14: drop-outs did not differ from participants | 3: not discussed  7: reports to have no information  13: no response rate given  14: no information available  19: funding yes but no apparent conflict of interest | 3: not discussed | 3: not discussed  19: funding yes but no apparent conflict of interest | 3: not discussed |

*Comments addressing where studies deviated from quality appraisal tool. Numbers in boxes correspond to question numbers.*

|  |  |  | Müller et al., 2019 | Entholt et al., 2018 | Oppedal & Isdoe, 2015 | Sierau et al., 2019 | Stotz et al., 2015 | Müller-Bamouth et al., 2016 | Geltman et al., 2005 | Huemer et al, 2013 | Porte & Torney-Purta, 1987 |
| --- | --- | --- | --- | --- | --- | --- | --- | --- | --- | --- | --- |
| Study design |  |  | cross | cross | cross | cross | cross | cross | cross | cross | cross |
| AXIS Quality |  |  | 17/20 | 15/20 | 18/20 | 19/20 | 14/20 | 16/20 | 19/20 | 16/20 | 13/20 |
| Risk of bias |  |  | medium | medium | low | low | medium | medium | low | medium | high |
| Introduction | 1 | Clear aims? | yes | yes | yes | yes | yes | yes | yes | yes | yes |
| Methods | 2 | Appropriate study design? | yes | yes | yes | yes | yes | yes | yes | yes | yes |
|  | 3 | Justified sample size? | no | no | no | no | no | no | no | no | yes |
|  | 4 | Clearly defined population? | yes | yes | yes | yes | yes | yes | yes | yes | yes |
|  | 5 | Appropriate sample population? | yes | yes | yes | yes | yes | yes | yes | no | yes |
|  | 6 | Process selects representative sample? | no | yes | yes | yes | yes | yes | yes | yes | yes |
|  | 7 | Addresses and categorises non-responders? | yes | no | yes | yes | no | no | yes | yes | no |
|  | 8 | Appropriate outcome variables? | yes | yes | yes | yes | yes | yes | yes | yes | yes |
|  | 9 | Valid instruments to measure outcomes? | yes | yes | yes | yes | yes | yes | yes | no | no |
|  | 10 | Statistical significance clear? | yes | yes | yes | yes | no | yes | yes | yes | yes |
|  | 11 | Methods described enable to be replicated? | yes | yes | yes | yes | yes | yes | yes | yes | no |
| Results | 12 | Basic data described adequately? | yes | yes | yes | yes | yes | yes | yes | yes | yes |
|  | 13 | Non-response bias concern? | yes | yes | no | no | yes | yes | no | yes | yes |
|  | 14 | Non-responders described? | yes | no | yes | yes | no | no | yes | yes | no |
|  | 15 | Results internally consistent? | yes | yes | yes | yes | yes | yes | yes | yes | yes |
|  | 16 | Results presented for all method analyses? | yes | no | yes | yes | yes | yes | yes | yes | yes |
| Discussion | 17 | Conclusion justified by results? | yes | yes | yes | yes | yes | yes | yes | yes | yes |
|  | 18 | Limitations discussed? | yes | yes | yes | yes | yes | yes | yes | yes | yes |
| Other | 19 | Funding or conflict of interest concern? | no | no | - | no | no | no | yes/no | no | - |
|  | 20 | Ethical approval or consent obtained? | yes | yes | yes | yes | no | yes | yes | yes | no |
| Comments |  |  | 3: not discussed  13: many facilities excluded (77%)  18: authors raise concers about selection bias and generalization | 3: not discussed  7: not discussed  10: not for all outcomes  13: no response rate given  14: not discussed  16: only descriptive statistics described, one correlation mentioned | 3: not discussed  19: No statement | 3: not discussed  13: response rate 75%  14: brief information given | 3: not discussed  7: no Information because of confidentiality  10: p-values mentioned but no level of significance described  13: no response reate given  14: not discussed  20: no statement | 3: not discussed  7: not discussed  13: no response rate given  14: not discussed | 3: not discussed  10: no significane level, but OR and CI  15: response rate 73%  19: funding yes but no apparent conflict of interest | 3: not discussed  5: too many predictors for such a small n  9: outcome YSR not validated or normed for this population / only correlations reported  13: only english speaking UMR included | 7: not discussed  9: self-developed questionaire not further described  10: p-values mentioned but no level of significance described  11: no information according self-developed questionaire  13: no response rate given  14: not discussed  19: no statement  20: no statement |

*Comments addressing where studies deviated from quality appraisal tool. Numbers in boxes correspond to question numbers.*
